# Supplementary material for: LPS O-antigen polysaccharide length impacts outer membrane permeability of enteric gram-negative bacteria
Source: mBio. 2025 Nov 5;16(12):e02518-25. doi: 10.1128/mbio.02518-25 (PMC12691688; doi:10.1128/mbio.02518-25)
Supplement: Supplemental Figures and Tables — Fig. S1-S4; Tables S1-S3. [file mbio.02518-25-s0001.pdf]

Supplemental Material

LPS O-antigen polysaccharide length impacts outer membrane permeability of enteric gram-negative bacteria

Kerrie L. May[a,b], Tatsuya Akiyama[b,c], Bella G. Parker[a,b], Minsu Kim[b,c], Marcin Grabowicz[a,b,d*]

Figures S1 to S4
Tables S1 to S3

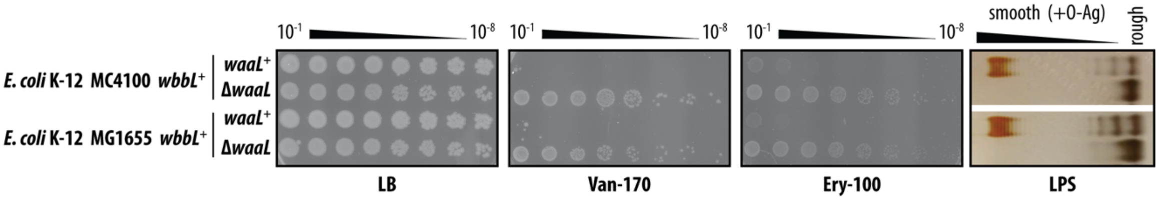

Fig. S1. Smooth LPS production in model *E. coli* K-12 strains leads to antibiotic sensitivity. Efficiency-of-plating antibiotic sensitivity assays using 10-fold serial dilutions of saturated culture. LPS profile of presented strains demonstrates smooth LPS production in *waaL*+ strains and isogenic Δ*waaL* strains that only produce rough LPS. Antibiotic concentrations are in μg/ml.

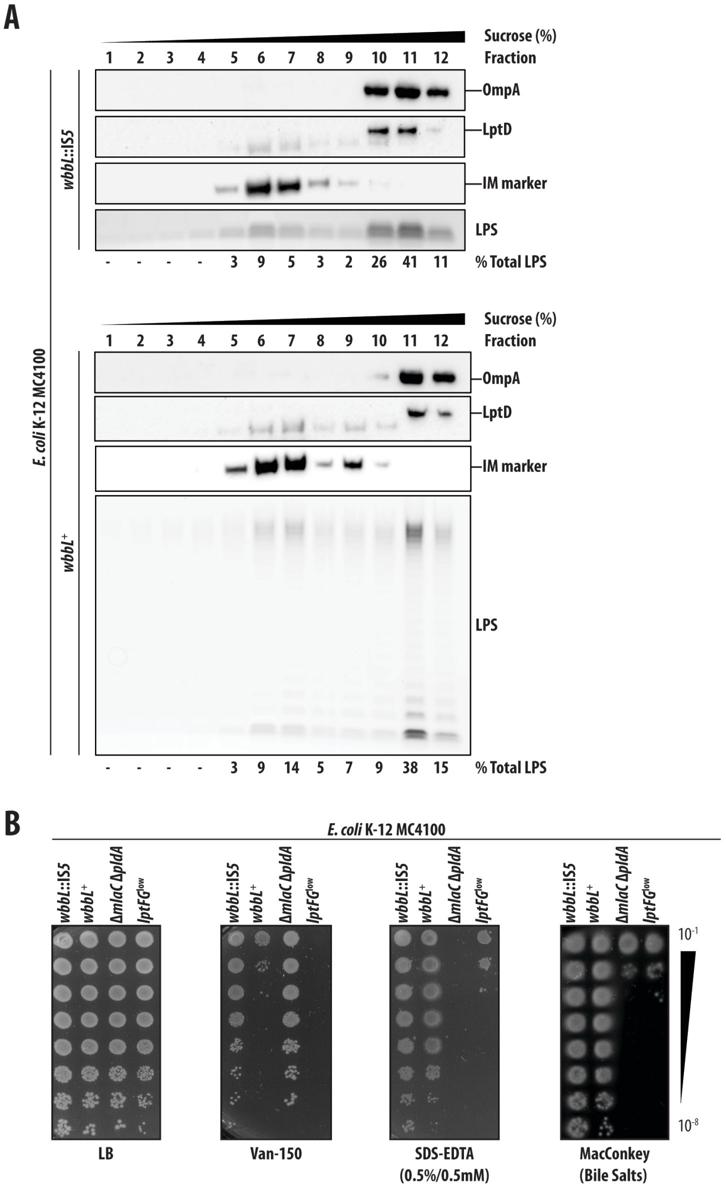

Fig. S2. Smooth LPS production in *E. coli* K-12 MC4100 has minimal impact on LPS transport and is phenotypically distinct from OM LPS deficiency or OM lipid asymmetry defects. (A) IM and OM were separated using sucrose gradient centrifugation and gradient fractions analyzed for composition. OM-containing fractions are identified by immunoblotting against OM β-barrel OMPs LptD and OmpA. IM-containing fractions are identified by immunoblotting for a 55-kDa IM protein marker. LPS from each fraction was isolated resolved by SDS-PAGE after samples were proteinase K-treated. LPS is visualized with Pro-Q Emerald 300. LPS bands were visualized by UV transillumination, and relative fluorescence intensities for all bands in each lane were determined with the Quantity One imaging software (Bio-Rad). **(B)** Efficiency-of-plating assays assessed detergent and bile salt sensitivity associated with disruptions to outer membrane asymmetry. Ten-fold serial dilutions of saturated cultures are presented. All data are representative of at least three independent experiments.

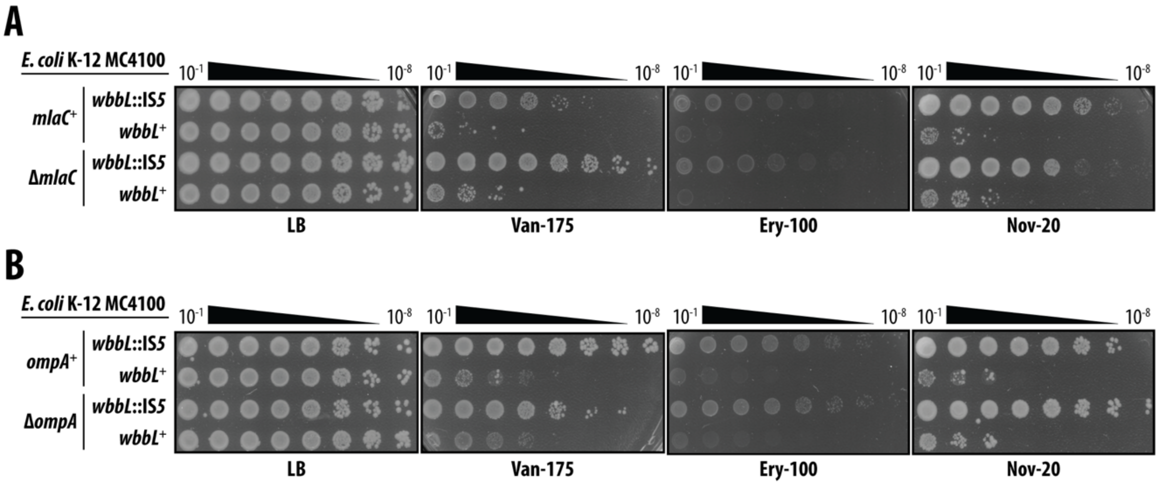

Fig. S3. Modulating OM lipid asymmetry or OM stiffness does not impact sensitivity of smooth LPS producing *E. coli* MC4100 to large scaffold antibiotics. (A) Efficiency-of-plating assays demonstrating that modulation of OM lipid asymmetry, through inactivation of maintenance of lipid asymmetry (Mla) pathway (Δ*mlaC*) does not alter *wbbL*$^{+}$ antibiotic sensitivity. **(B)** Efficiency-of-plating assays demonstrating that decreasing OM stiffness, through removal of OmpA (Δ*ompA*) does not alter *wbbL*$^{+}$ antibiotic sensitivity. Ten-fold serial dilutions of saturated cultures are shown.

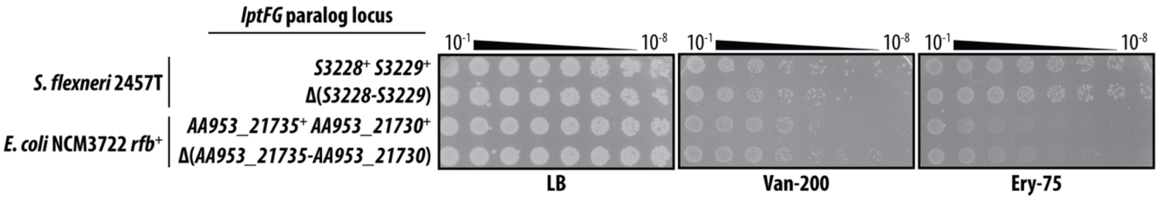

Fig. S4. The LpF and LptG paralogs of *S. flexneri* 2457T and *E. coli* K-12 NCM3722 that are missing in *E. coli* MC4100 do not impact the OM antibiotic barrier when smooth LPS is produced. Efficiency-of-plating assays to assess antibiotic sensitivity in *S. flexneri* 2457T producing or lacking *lptFG* paralogs (genomic loci *S3228* and *S3229*) and in *E. coli* K-12 NCM3722 producing or lacking *lptFG* paralogs (genomic loci *AA953_21735* and *AA953_21730*). Ten-fold serial dilutions of saturated cultures are shown. Antibiotic concentrations are in μg/ml.

Table S1: Strains used in this study

Strain	Genotype	Reference
MC4100	*E. coli* K-12; F^- *araD139* Δ(*arg-lac*)*U169 rpsL150 relA1 flbB5301 deoC1 ptsF25 thi wbbL::*IS*5*	(1)
MG1655	*E. coli* K-12; F^- λ^- *ilvG*$^-$ *rfb*-50 *rph*-1 *wbbL::*IS*5*	(2)
NCM3722	*E. coli* K-12; λ^+ *gal*$^+$ *eut*$^+$ *pyrE*$^+$ *ilvG*$^+$ *rpoS33Am glnV(SupAm) evgA::IS1 Δrfb*	(3)
2457T	*S. flexneri; wildtype*	ATCC 700930
MG873	MC4100 *wbbL*$^+$	This study
MG1230	MG1655 *wbbL*$^+$	This study
KM611	MC4100 [pDSW204]	This study
KM607	MC4100 *wbbL*$^+$ [pDSW204]	This study
KM613	MC4100 [pDSW204-uppS]	This study
KM609	MC4100 *wbbL*$^+$ [pDSW204-uppS]	This study
KM850	MC4100 Δ*waaL*	This study
MG902	MC4100 Δ*waaL wbbL*$^+$	This study
MG1231	MG1655 *wbbL*$^+$ Δ*waaL*	This study
KM1090	MC4100 Δ*ompA*::*kan hisG*::Tn*10*	This study
KM1091	MC4100 Δ*ompA*::*kan wbbL*$^+$ *hisG*::Tn*10*	This study
KM1088	MC4100 Δ*mlaC hisG*::Tn*10*	This study
KM1089	MC4100 Δ*mlaC wbbL*$^+$ *hisG*::Tn*10*	This study
MG475	MC4100 Δ*mlaC* Δ*pldA*	This study
KM1033	MC4100 *bla Para::lptFG hisG*::Tn*10*	(4) , this study
KM1034	MC4100 *bla Para::lptFG wbbL*$^+$ *hisG*::Tn*10*	(4) , this study
MG1708	MC4100 [pET23/42::*lptD4213*]	This Study
MG1709	MC4100 *carB*::Tn*10* Δ*lptD*::*kan* [pET23/42::*lptD4213*]	This study
MG1322	MC4100 *wbbL*$^+$ *wzzB*(Q205Am)	This Study
KM1045	MC4100 Δ*wzzB::kan hisG*::Tn*10* [pET17b]	This study
KM1046	MC4100 Δ*wzzB::kan hisG*::Tn*10* [pET17b-*wzz*$_{SF}$]	This study
KM1049	MC4100 Δ*wzzB::kan hisG*::Tn*10* [pET17b-*wzz*$_{SF\text{-}K267N}$]	This study
KM1048	MC4100 Δ*wzzB::kan hisG*::Tn*10* [pET17b-*wzz*$_{SF}$::*wzz*$_{ST}$]	This study
KM1042	MC4100 Δ*wzzB::kan hisG*::Tn*10* [pWSK29]	This study
KM1043	MC4100 Δ*wzzB* [pWSK29-*wzz*$_{ST}$]	This study
KM1044	MC4100 Δ*wzzB* [pWSK29-*wzz*$_{fepE}$]	This study
MG1232	NCM3722 *rfb*$^+$ *wca*$^+$ *cps*$^+$	This study
KM1061	2457T Δ*wzz*$_{SF}$*::kan*	This study
MG4748	2457T Δ*rmlD::kan*	This study

Table S2: Plasmids used in this study

Plasmid	Description	Reference/ Source
pBAD18	Cloning vector for arabinose-inducible expression, Amp^R	(5)
pDSW204	vector	(6)
pUPPS	pDSW204::uppS	(6)
pCP20	FLP recombinase plasmid, Cam^R Amp^R	(7)
pCD77	pET17b	(8)
pCD78	pET17b::wzz_{SF}	(8)
pCD108	pET17b::$wzz_{SF-K267N}$	(8)
pCD106	pET17b::wzz_{SF}-wzz_{ST}	(8)
pCD80	pET17b::wzz_{ST}	(8)
pBBR1MCS	cloning vector, Cam^R	(9)
pBBR1::$waaL_{SF}$	contains cloned chromosomal DNA fragment from Shigella flexneri 2457T (nt 400036-4001692) which encodes $waaL^{SF}$, with gene expression driven from native promoter	This study

Table S3: Oligonucleotides used in this study

Name	Sequence (5'-3')	Note
wzzB_UP_F	tgt gct gca agg cga tta agt tgg gtc caa ccg act atg atc c	Upstream homology region for Gibson assembly with pKD4 Kan-cassette; template for Lambda Red allelic exchange mutagenesis
wzzB_UP_R	cga agc agc tcc agc cta cac aat cca tag ttt agc gat aat ccc	Downstream homology region for Gibson assembly with pKD4 Kan-cassette; template for Lambda Red allelic exchange mutagenesis
wzzB_DOWN_F	agg aac taa gga gga tat tca tat gcg taa tta caa cgc gaa gta ata tta ttg	Upstream homology region for Gibson assembly with pKD4 Kan-cassette; template for Lambda Red allelic exchange mutagenesis
wzzB_DOWN_R	taa caa ttt cac aca gga aac agc ttg aag ttt gcc acg tcc	Upstream homology region for Gibson assembly with pKD4 Kan-cassette; template for Lambda Red allelic exchange mutagenesis
rmlD_pBBR1MCS_Fv2	tgt gct gca agg cga tta agt tgg gtg cag tgc act ggt agc	Upstream homology region for Gibson assembly with pKD4 Kan-cassette; template for Lambda Red allelic exchange mutagenesis
rmlD_pKD4kan_R	cga agc agc tcc agc cta cac aat cta ctg acg gcc ctc ata g	Downstream homology region for Gibson assembly with pKD4 Kan-cassette; template for Lambda Red allelic exchange mutagenesis
rmlD_pKD4kan_F	agg aac taa gga gga tat tca tat gac gac cac ggc aat tta aca	Upstream homology region for Gibson assembly with pKD4 Kan-cassette; template for Lambda Red allelic exchange mutagenesis
rmlD_pBBR1MCS_R	taa caa ttt cac aca gga aac agc ttc aac agc tac gtc aaa aac ttc a	Upstream homology region for Gibson assembly with pKD4 Kan-cassette; template for Lambda Red allelic exchange mutagenesis

SUPPLEMENTAL REFERENCES

1. M. J. Casadaban, Transposition and fusion of the lac genes to selected promoters in Escherichia coli using bacteriophage lambda and Mu. *J. Mol. Biol.* **104**, 541–555 (1976).

2. M. S. Guyer, R. R. Reed, J. A. Steitz, K. B. Low, Identification of a Sex-factor-affinity Site in E. coli as . *Cold Spring Harb. Symp. Quant. Biol.* **45**, 135–140 (1981).

3. E. Soupene, *et al.*, Physiological Studies of Escherichia coli Strain MG1655: Growth Defects and Apparent Cross-Regulation of Gene Expression. *J. Bacteriol.* **185**, 5611–5626 (2003).

4. Z. Yao, R. M. Davis, R. Kishony, D. Kahne, N. Ruiz, Regulation of cell size in response to nutrient availability by fatty acid biosynthesis in Escherichia coli. *Proc. Natl. Acad. Sci. U.S.A.* **109**, E2561-8 (2012).

5. L. M. Guzman, D. Belin, M. J. Carson, J. Beckwith, Tight regulation, modulation, and high-level expression by vectors containing the arabinose PBAD promoter. *J. Bacteriol.* **177**, 4121–4130 (1995).

6. M. A. Jorgenson, K. D. Young, Interrupting Biosynthesis of O Antigen or the Lipopolysaccharide Core Produces Morphological Defects in Escherichia coli by Sequestering Undecaprenyl Phosphate. *J. Bacteriol.* **198**, 3070–3079 (2016).

7. K. A. Datsenko, B. L. Wanner, One-step inactivation of chromosomal genes in Escherichia coli K-12 using PCR products. *Proc. Natl. Acad. Sci. U.S.A* **97**, 6640–6645 (2000).

8. C. Daniels, R. Morona, Analysis of Shigella flexneri Wzz (Rol) function by mutagenesis and cross-linking: Wzz is able to oligomerize. *Mol. Microbiol.* **34**, 181–194 (1999).

9. M. E. Kovach, *et al.*, Four new derivatives of the broad-host-range cloning vector pBBR1MCS, carrying different antibiotic-resistance cassettes. *Gene* **166**, 175–176 (1995).

10. A. Ducret, E. M. Quardokus, Y. V. Brun, MicrobeJ, a tool for high throughput bacterial cell detection and quantitative analysis. *Nat. Microbiol.* **1**, 16077 (2016).

11. J. Schindelin, *et al.*, Fiji: an open-source platform for biological-image analysis. *Nat. Methods* **9**, 676–682 (2012).

12. K. M. Lehman, K. L. May, J. Marotta, M. Grabowicz, Genetic analysis reveals a robust and hierarchical recruitment of the LolA chaperone to the LolCDE lipoprotein transporter. *mBio* **15**, e03039-23 (2024).

13. T. Wu, *et al.*, Identification of a protein complex that assembles lipopolysaccharide in the outer membrane of Escherichia coli. *Proc. Natl. Acad. Sci. U.S.A.* **103**, 11754–11759 (2006).
